# Down-regulation of TUFM impairs host cell interaction and virulence by *Paracoccidioides brasiliensis*

**DOI:** 10.1038/s41598-019-51540-y

**Published:** 2019-11-20

**Authors:** Caroline Maria Marcos, Gabrielle Tamer, Haroldo Cesar de Oliveira, Patricia Akemi Assato, Liliana Scorzoni, Claudia Tavares Santos, Junya de Lacorte Singulani, Julhiany de Fátima da Silva, Rodrigo de Almeida, Ana Carolina Alves de Paula e Silva, Rosangela Aparecida Moraes da Silva, Cleverton Roberto de Andrade, Diana Patricia Tamayo, Angela Maria Lopez, Natália Moreira Barbosa, Cleslei Fernando Zanelli, Orville Hernandez- Ruiz, Juan G. McEwen, Maria José Soares Mendes-Giannini, Ana Marisa Fusco-Almeida

**Affiliations:** 1 Faculdade de Ciências Farmacêuticas, UNESP – Univ Estadual Paulista, Campus Araraquara, Departamento de Análises Clínicas, Laboratório de Micologia Clinica, São Paulo, Brazil; 2 Faculdade de Odontologia, UNESP – Univ Estadual Paulista, Campus Araraquara, Departamento de Fisiologia e Patologia, São Paulo, Brazil; 3 Unidad de Biología Celular y Molecular, Corporación para Investigaciones Biológicas (CIB), Medellín, Colombia; 4 Faculdade de Ciências Farmacêuticas, UNESP – Univ Estadual Paulista, Campus Araraquara, Departamento de Ciências Biológicas, Laboratório de Biologia Molecular e Celular de Microrganismos, São Paulo, Brazil; 5 Grupo de Investigación MICROBA -Escuela de Microbiología, Universidad de Antioquia, Medellín, Colombia; 6 Facultad de Medicina, Universidad de Antioquia, Medellín, Colombia; 7 Instituto Carlos Chagas, Fundação Oswaldo Cruz (Fiocruz), Curitiba, Brazil; 8 Institute of Science and Technology, São Paulo State University (UNESP), São José dos Campos, Brazil

**Keywords:** Fungal host response, Infection

## Abstract

The genus *Paracoccidioides* consist of dimorphic fungi geographically limited to the subtropical regions of Latin America, which are responsible for causing deep systemic mycosis in humans. However, the molecular mechanisms by which *Paracoccidioides* spp. causes the disease remain poorly understood. *Paracoccidioides* spp. harbor genes that encode proteins involved in host cell interaction and mitochondrial function, which together are required for pathogenicity and mediate virulence. Previously, we identified TufM (previously known as EF-Tu) in *Paracoccidioides brasiliensis* (PbTufM) and suggested that it may be involved in the pathogenicity of this fungus. In this study, we examined the effects of downregulating PbTUFM using a silenced strain with a 55% reduction in PbTUFM expression obtained by antisense-RNA (aRNA) technology. Silencing PbTUFM yielded phenotypic differences, such as altered translation elongation, respiratory defects, increased sensitivity of yeast cells to reactive oxygen stress, survival after macrophage phagocytosis, and reduced interaction with pneumocytes. These results were associated with reduced virulence in *Galleria mellonella* and murine infection models, emphasizing the importance of PbTufM in the full virulence of *P. brasiliensis* and its potential as a target for antifungal agents against paracoccidioidomycosis.

## Introduction

The genus *Paracoccidioides* comprises several species of thermodimorphic fungi, including *Paracoccidioides brasiliensis*, *P. americana, P. restrepiensis, P. venezuelensis*, and *P. lutzii*, which are the etiologic agents of paracoccidioidomycosis (PCM). 80% of PCM cases in Latin America were reported in Brazil^1–4^. At 36 °C *Paracoccidioides* spp. grow as yeast cells *in vitro* and in vertebrate tissues, whilst at 23 °C they grow as mycelia *in vitro* and in the soil. Entry into the host occurs mainly via the inhalation of spores or propagules, which initially establish in the lungs and can then disseminate through the body causing damage to other internal organs^5,6^.

*Paracoccidioides* spp. has mechanisms that enable its adhesion and invasion to several cell types, that contributes to infect and colonize host tissues. The adhesion process, whether it be directly to cells or to the surrounding macromolecular matrix, is important for the invasion, growth, persistence, and pathogenesis of *Paracoccidioides* spp.^7,8^. To colonize the site of infection, the fungus expresses several surface proteins which establish contact with the host cell or extracellular matrix (ECM) and may induce the cytoskeletal rearrangement of host cells^9,10^.

During the colonization and invasion of the host, the pathogen uses various mechanisms to subvert host immune defenses^11,12^. After the inhalation of conidia, *Paracoccidioides* can be phagocytized by several types of cell, including macrophages which have a depleted nutrient environment and produce reactive oxygen species (ROS) and reactive nitrogen species (RNS) with antimicrobial activities. However, the fungus triggers defensive strategies in order to survive under such conditions^13,14^.

*Paracoccidioides* spp. possesses multiples attributes allowing them to adhere, colonize, disseminate, and adapt to various sites in the human body. Therefore, it is essential that the molecules involved in these processes be identified to improve our understanding of *Paracoccidioides* spp. virulence and PCM pathogenesis. Moreover, molecules that reduce the virulence of pathogens may prove a promising alternative to conventional antimicrobials^15^.

Previously, Marcos *et al*.^16^ identified the *P. brasiliensis* TufM protein, and used recombinant PbTufM and its respective polyclonal antibody to demonstrate its surface localization and its importance in interactions with pneumocytes and ECM components, such as fibronectin and plasminogen. These data suggest that PbTufM may be suitable for direct biological analysis by generating silenced or knockout isolates to evaluate its effects on the fitness of *P. brasiliensis*.

The steps involved in translation are the same in both the mitochondrial and cytosolic systems, however the translation factors involved are different^17^. Unlike cytosolic translation, the processes underlying the protein synthesis in mitochondria still need to be better elucidated. The majority of ribosomal proteins, tRNA synthetases, and factors involved in ribosome assembly and mitochondrial translation, are codified in the nucleus, synthesized in the cytoplasm, and then delivered to the mitochondria^18^. EF-Tu plays an fundamental role in translation conferring charged tRNAs to the ribosomal A-site during peptide chain elongation enabling protein synthesis^19^. Although orthologues of the *S. cerevisiae* mitochondrial EF-Tu, EF-G, and RF1 have been identified in the *P. brasiliensis* transcriptome, no sequences have been found corresponding to functional EF-Ts^20,21^.

The absence of protein synthesis due to the deletion of mitochondrial elongation factor (TufM) prevented growth on a respiratory substrate in *S. cerevisiae*. Alterations to mitochondrial EF-Tu affect multiple cellular networks and pathways, and consequently cell physiology and homeostasis^22^. The *vad1∆ Cryptococcus neoformans* mutant has decreased TUF1 (an EF-Tu homologue) expression and has been shown to have impaired growth on a respiratory medium with a glycerol carbon source. TUF1 overexpression improves any aspects of this phenotype related to mitochondrial function, such as the ability to grow on a respiratory medium^23^.

To date, the function of PbTufM has not been determined with respect to cell biology and effects of PbTufM on the virulence of *P. brasiliensis*. Here, we silenced the TUFM gene using antisense-RNA (aRNA), which has a well-established methodology for use in this fungus^24,25^. Additionally, we studied the effects of PbTufM on *P. brasiliensis* growth and viability, its ability to interact with pneumocytes, and its virulence in alternative invertebrate (*Galleria mellonella*) and murine models. Our results indicated that the silenced strain, *PbTufM* aRNA, retained its morphology but had respiratory defects and increased sensitivity to oxidative stress. In relation to fungal fitness, this phenotype likely reflects the reduced ability of *P. brasiliensis* to interact with host cells.

## Results

### TufM is localized in the mitochondria of *P. brasiliensis*

**Figure 1:**
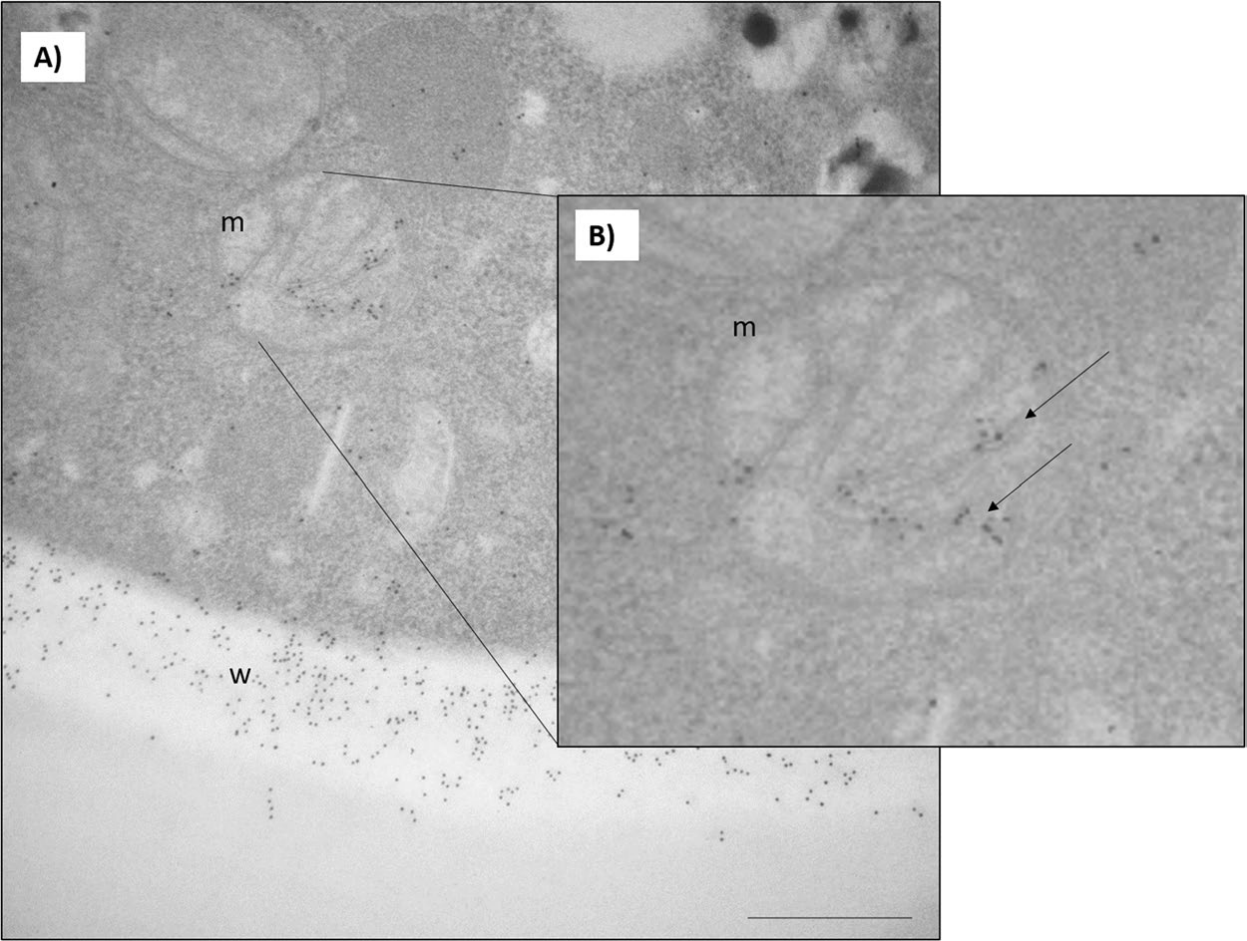
Immunogold labeling of the TufM protein of *P. brasiliensis*. m: mitochondria, w- cell wall. Bars = 1 µm. (**A**) Gold particles that correspond to PbTufM are observed in the fungus cell (arrows). (**B**) The highlighted square points to the PbTufM in the mitochondria.

Previously, Marcos *et al*.^16^ reported the cell surface and cytoplasmic localization of TufM. Using immunoelectron microscopy we verified that TufM is present in the mitochondria, a finding confirmed by the immunogold labeling of this organelle (Fig. 1).

### Suppression of PbTUFM expression using an aRNA plasmid and ATMT

**Figure 2:**
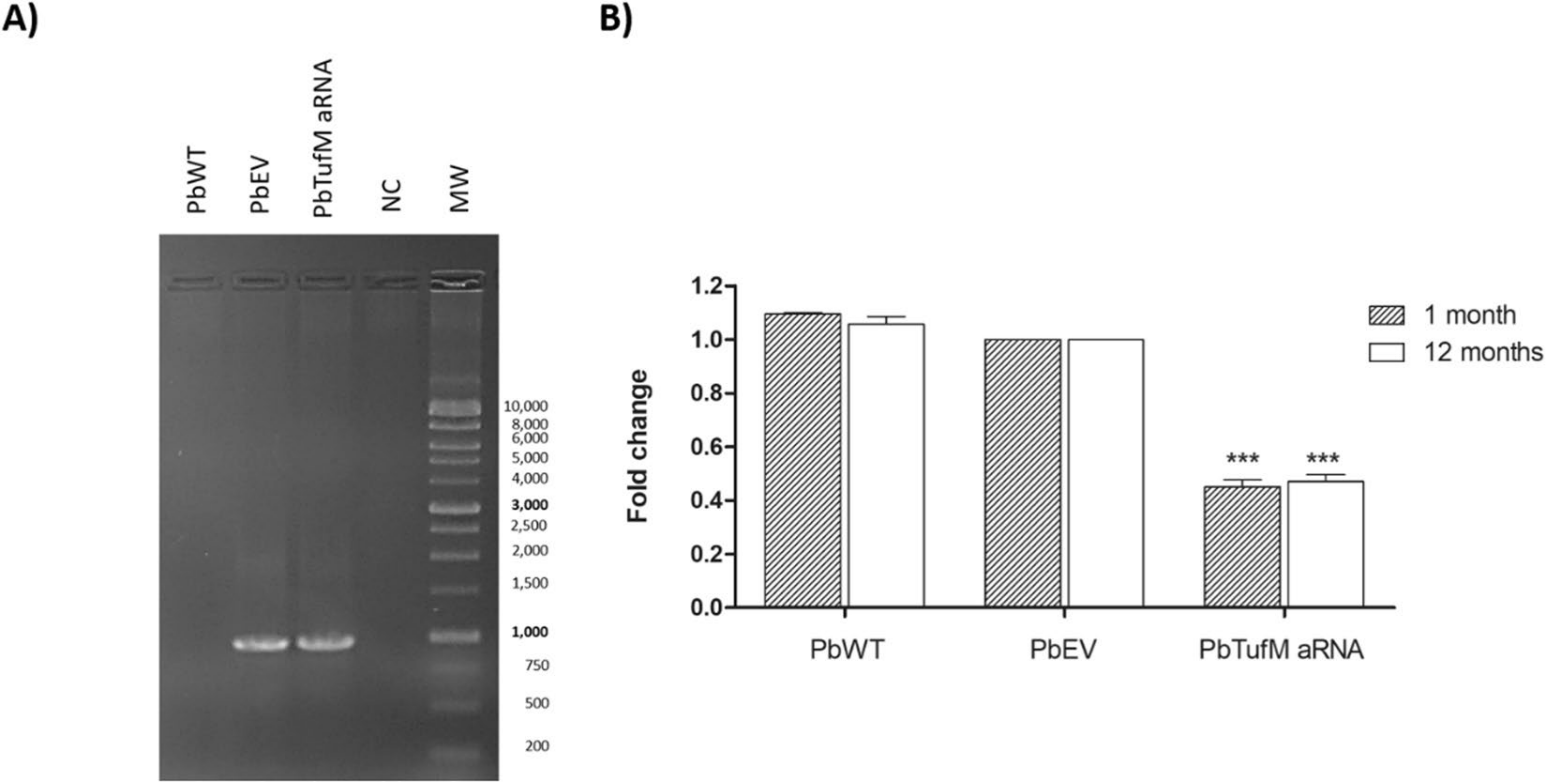
Obtaining *P. brasiliensis PbTufM* aRNA isolate. (**A**) Agarose gel electrophoresis to confirm the transference of *HPH* gene by ATMT to PbEV (PbWT transformed with empty vector) and *PbTufM* aRNA (PCR amplification yielded a 1,000 bp internal fragment), NC: negative control of PCR reaction, MW: 1Kb DNA ladder (GeneDireX). (**B**) Expression levels of PbTUFM assessed by Real-Time PCR in PbWT, PbEV, and *PbTufM* aRNA yeast cells after being subcultured for 1 and 12 months (gene expression levels obtained by RT-PCR were normalized to the level of expression of the internal reference, β-tubulin; ***p < 0.0001 compared with PbWT and PbEV.

To verify the consequences of TUFM downregulation in *P. brasiliensis*, we used aRNA and *Agrobacterium tumefaciens*-mediated transformation (ATMT). aRNA corresponding to the homologous sequences of the PbTUFM exon were constructed for genomic integration by ATMT. PbTUFM downregulated, hygromycin-resistant transformants of *P. brasiliensis* wild-type Pb18 were selected and successive subcultured in a non-selective and selective medium with hygromycin (HPH) for three consecutive rounds. The presence of the hygromycin cassette in the genomic DNA of a *P. brasiliensis* transformant, named *PbTufM* aRNA, was confirmed using PCR (1,000 bp, Fig. 2A) with HPH-specific primers, and the transformant was selected for gene expression analysis. The levels of TUFM mRNA transcript in *PbTufM* aRNA were measured after 1 month and over 12 months (without statistical differences for the same isolate comparing the evaluated times) of subculture using RT-qPCR and were found to be 64–58% lower than in PbWT (Pb18; wild type-isolate) and 55–53% than in PbEV (empty vector), respectively (Fig. 2B).

### *PbTufM* aRNA has a morphology typical of *P. brasiliensis* and decreased ability to utilize a non-fermentable carbon source for growth

**Figure 3:**
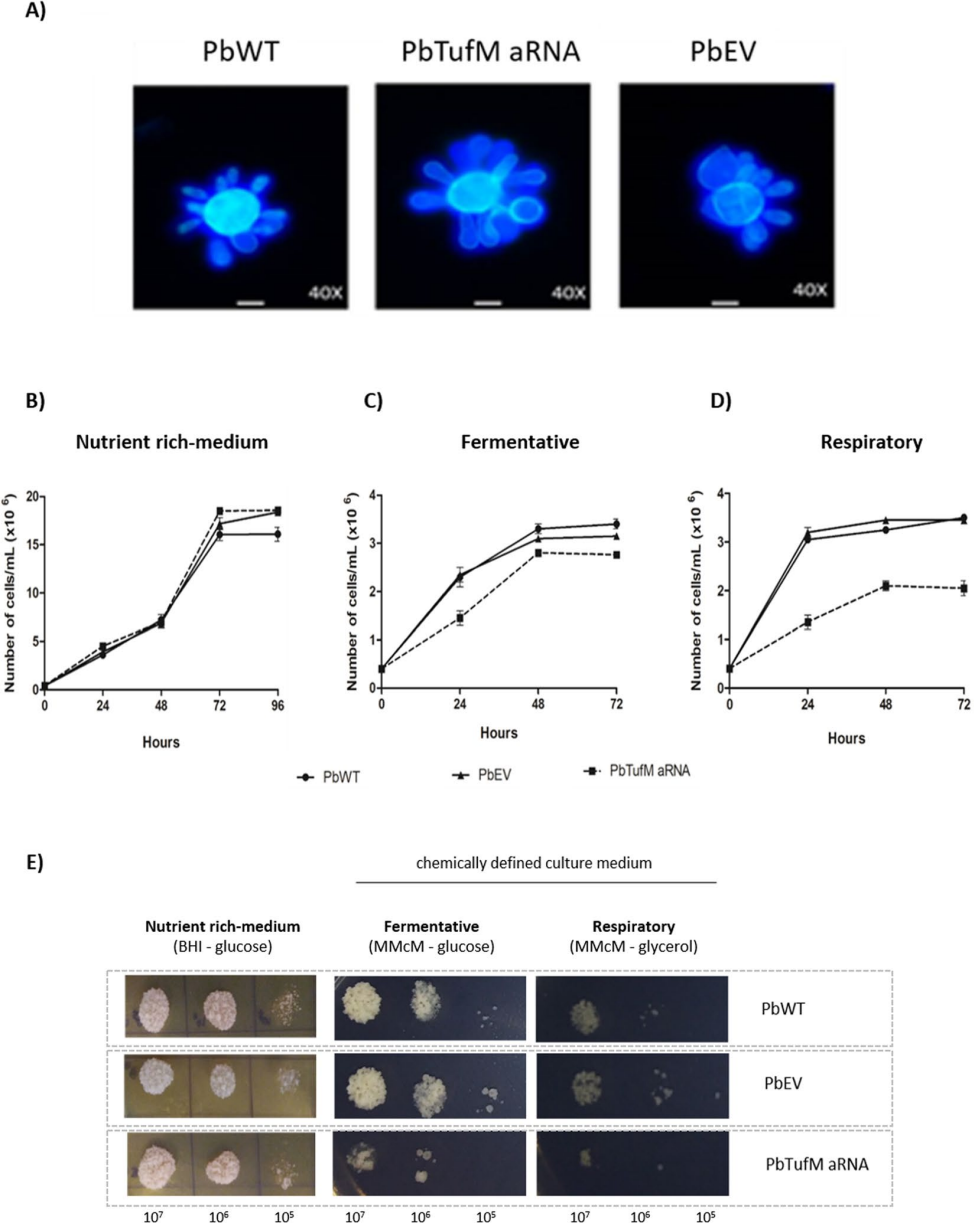
Silencing of PbTUFM and consequences for *P. brasiliensis* yeast cell growth, viability and morphology. Cellular morphology of exponentially grown yeast cells from PbWT, PbEV, and *PbTufM* aRNA stained with Calcofluor white and visualized by fluorescence microscopy. Magnification of 40× and white bars correspond to 20 μm (**A**). Growth curves and viability of *P. brasiliensis* isolates PbWT, PbEV, and *PbTufM* aRNA were evaluated by counting of yeasts by optical microscopy using Neubauer chamber and spotting in BHI medium (nutrient rich-mediun) (**B**,**E**), McMM medium with glucose (**C**,**E**) and McMM medium with glycerol (**D**,**E**).

We analyzed the effects of PbTUFM knockdown by comparing the viability, growth rate, and yeast morphology of the *PbTufM* aRNA, PbWT, and PbEV strains. Microscopic observation showed that *PbTufM* aRNA cells were not phenotypically different in fungal morphology than PbWT and PbEV cells. *PbTufM* aRNA cells had a normal yeast morphology consisting of a single large central cell with multiple buds attached, and no differences between isolates (Fig. 3A).

*PbTufM* aRNA showed no growth defects under rich fermentable conditions (BHI medium with glucose; Fig. 3B,E), however there was a slight decrease in growth in the other fermentable medium (MMcM with glucose; Fig. 3C,E), and a greater decrease under respiratory conditions (MMcM with glycerol; Fig. 3D,E). Additionally, the growth of *PbTufM* aRNA cells (12.95 h) in BHI was comparable to that of PbWT (13.5 h) and PbEV (13.05 h) cells. The silenced cells showed a small increase in doubling time when grown in MMcM with glucose (*PbTufM* aRNA [17.05 h], PbWT [15.5 h], and PbEV [16 h]), and a larger increase in doubling time when grown under respiratory conditions (*PbTufM* aRNA [20.4 h], PbWT [15.45 h], and PbEV [15.4 h]). These data suggest that the silenced strain has an impaired respiratory capacity (Table 1).

**Table 1:** Growth rate of PbWT, PbEV and *PbTufM* aRNA in different carbon sources.

Strain	Carbon source	Doubling time* (hours)
PbWT	Glucose, aminoacids and polypeptides (BHI with glucose)	13.50 ± 0.287
PbEV		13.05 ± 0.092
*PbTufM* aRNA		12.95 ± 0.055
PbWT	Glucose (MMcM with glucose)	15.50 ± 0.304
PbEV		16.00 ± 0.169
*PbTufM* aRNA		17.05 ± 0.205
PbWT	Glycerol (MMcM with glycerol)	15.45 ± 0.028
PbEV		15.40 ± 0.148
*PbTufM* aRNA		20.40 ± 0.565

*Average of three independent experiments with standard deviation.

### Decreased TUFM levels alter translation rates in *P. brasiliensis*

**Figure 4:**
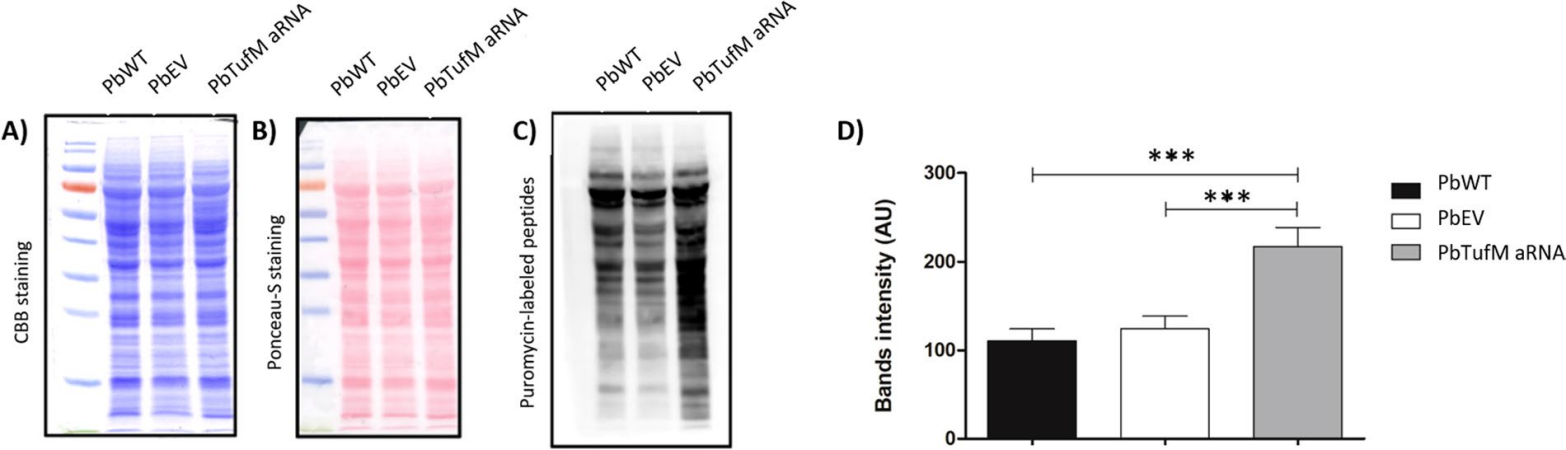
Effect in global translation rate due to PbTufM reduction. The rate of translation in PbWT, PbEV, and *PbTufM* aRNA was measured as the rate of incorporation of puromycin into newly synthesized polypeptides (by puromycin-labeled assay). (**A**) Gel stained with CBB (Coomassie Brilliant Blue) to demonstrated equal protein loading, (**B**) Ponceau-S staining showing total proteins levels and (**C**) western blotting with anti-puromycin antibody, (**D**) relative band intensity of newly synthesized proteins with puromycin incorporated in all strains. *p* < 0.0001 when *PbTufM* aRNA compared with PbEV and PbWT.

By labeling translated proteins with puromycin, we observed that *PbTufM* aRNA cells incorporate more puromycin when compared to PbWT (1.98-fold) and PbEV (1.75-fold) cells (Fig. 4C,D), indicating that decreased levels of TufM altered global translation rate in *P. brasiliensis*. Since the incorporation of puromycin into a growing peptide chain during translation leads to the termination of peptide elongation, the silenced strain may have slower elongation allowing more puromycin to be incorporated into the polypeptides.

### TUFM silencing reduces the levels of cell surface and cytosolic TufM in *P. brasiliensis*

Since it has been reported that TufM is present on the cell surface and in the cytosol of *P. brasiliensis*^16^, we examined the effects of TUFM silencing on its localization by using permeabilized (total protein) and non-permeabilized (surface protein) yeast cells.

**Figure 5:**
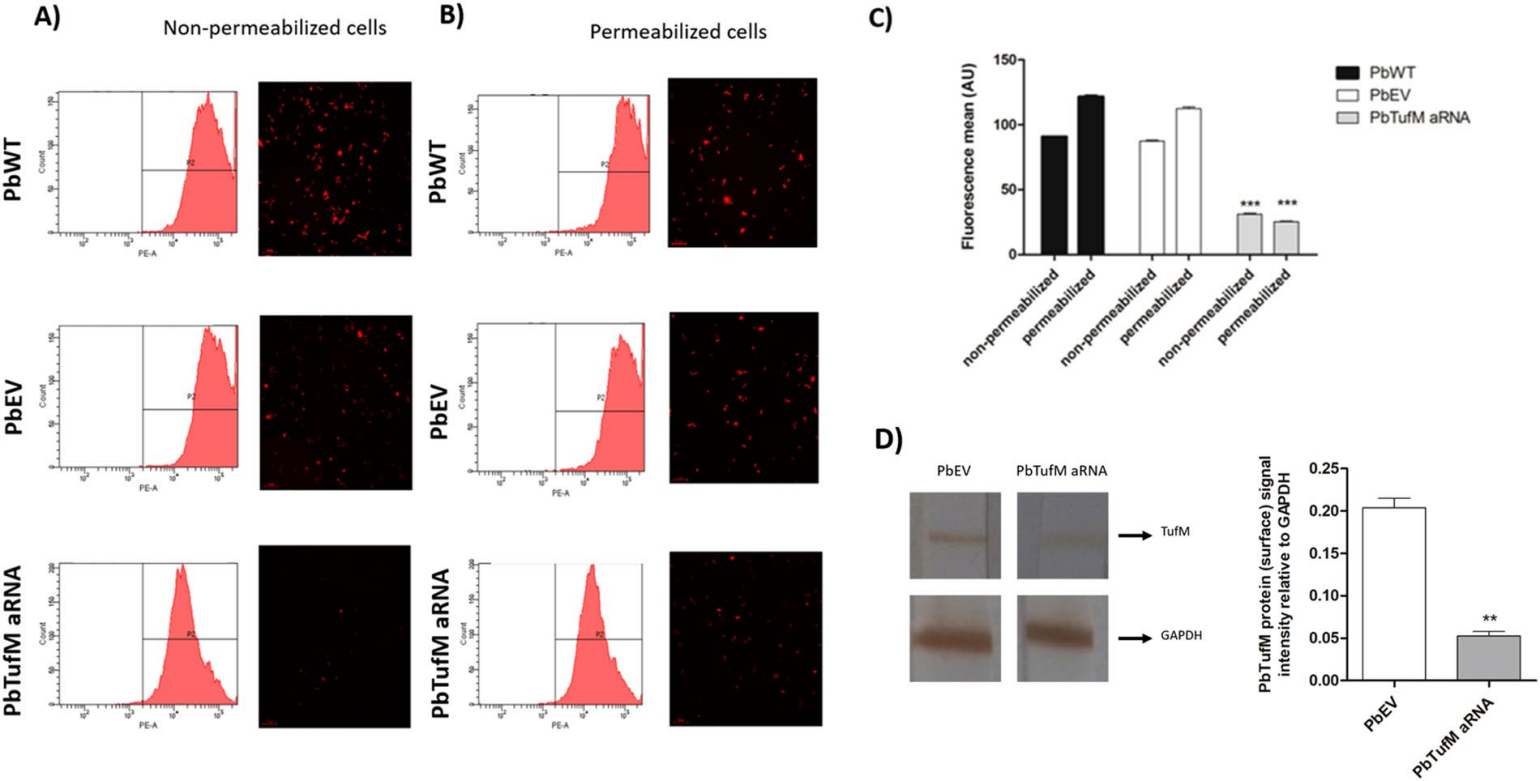
Downregulation of PbTUFM decreases TufM protein on cell surface and in cytosol. Flow-cytometry histograms and immunofluorescent microscopy images of PbWT, PbEV, and *PbTufM* aRNA non-permeabilized yeast cells (**A**) and permeabilized yeast cells (**B**) that were incubated with anti-PbTufM polyclonal serum (1:10). Total of 10,000 cells were analyzed per experiment. (**C**) Quantification estimate of TufM levels presented as mean of fluorescence. ***p < 0.001 when *PbTufM* aRNA was compared with PbEV and PbWT. D) Expression levels of TufM in *P. brasiliensis* surface. One representative blot is shown of three independente experiments. *Bar chart* shows quantification of protein levels compared to GAPDH control in each strain. **p < 0.05.

Flow-cytometry showed that both permeabilized and non-permeabilized *PbTufM* aRNA cells had a lower fluorescence intensity than PbWT and PbEV cells, with reductions of approximately 90.3% and 64.4%, respectively, when compared to PbEV cells (PbEV showed no difference in fluorescence compared to PbWT) (Fig. 5C). The TufM fluorescence intensity measurements were concordant with the TufM fluorescence visualized using microscopy (Fig. 5A,B).

Moreover, western blot analysis of the surface protein extracts of PbEV and *PbTufM* aRNA with anti-PbTufM (normalized using GAPDH as a control), revealed that the level of surface TufM was reduced 3.88-fold in the silenced strain. Taking the level of TufM expression in PbEV to be 100%, the reduction in *PbTufM* aRNA was approximately 25.7% (Fig. 5D), reinforcing above data which showed that the reduction in TufM protein levels also occurs on the cell surface.

### TUFM knockdown influences the ability of *P. brasiliensis* to interact with pneumocytes

**Figure 6:**
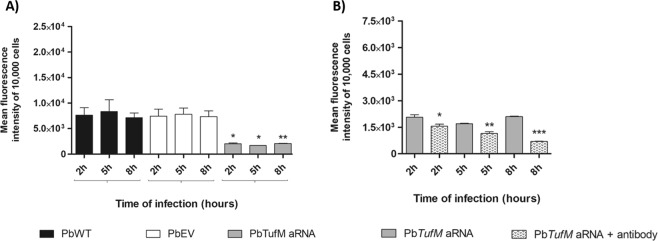
*PbTufM* aRNA has impairment in the interaction with pneumocytes. (**A**) The yeast-host cells interaction was evaluated measuring the fluorescence of infected pneumocytes in systems where fungal cells were labeled with CFSE. *p < 0.05; p < 0.005 when comparing *PbTufM* aRNA with PbWT and PbEV at 8 and 24 h, respectively. (**B**) The coating of *PbTufM* aRNA with anti-Pb*TufM* antibody has the potential to influence the pathogen-pneumocytes interaction. Interaction profile of *PbTufM* aRNA strain with pneumocytes after blocking of PbTufM surface protein with the treatment with anti-PbTufM polyclonal antibody. After treatment with the antibody the yeast cells were labelled with CFSE and then used in the A549 infection. The yeast-host cells interaction was evaluated measuring the fluorescence of infected pneumocytes in systems where fungal cells were labeled with CFSE. *p < 0.05; **p < 0.005; p < 0.0001.

TufM is expressed on the cell surface of *P. brasiliensis*, and it has previously been shown that treating *P. brasiliensis* with anti-TufM specific antibodies reduced its interactions with pneumocytes, demonstrating that cell surface TufM is important for interacting with host cells^16^. We investigated the ability of *P. brasiliensis* to interact with pneumocytes and cause infection by adhering to and invading host cells^26^. Compared to PbWT and PbEV, *PbTufM* aRNA cells had reduced adhesion and internalization in pneumocytes at 2, 5, 8 h post-infection by 3.6-fold, 4.5-fold, 3.5-fold respectively (Fig. 6A).

Moreover, the inhibition of the *PbTufM* aRNA -pneumocyte interaction was considerably more pronounced when cell surface PbTufM was blocked using specific anti-PbTufM antibodies, with reductions of 1.3-fold, 1.4-fold, 2.9-fold at 2, 5, 8 h post-infection, respectively (Fig. 6B). These data suggest that PbTufM is important in the pathogenic host cell interactions of *P. brasiliensis*.

### PbTUFM silencing increases sensitivity to oxidative stress and decreases the survival of *P. brasiliensis* against macrophages

**Figure 7:**
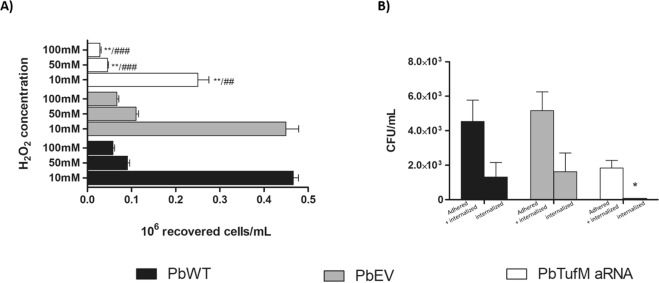
Viability of *PbTufM* aRNA was affected in oxidative stress condition. (**A**) The yeast viability of PbWT, PbEV, *PbTufM* aRNA treated with different concentrations of H_2_O_2_ for 1 h by CFU assay; **p < 0.005 when compared to PbWT, ^##^p < 0.005 when compared to PbEV and ^###^p < 0.0001 when compared to PbEV. (**B**) Interaction assay of *P. brasiliensis* and macrophage cells. Yeast cell viability after 2 h of macrophage infection (total infection- adhesion and invasion process; and invasion process) was evaluated based on the number of recovered CFU. *p < 0.005 when compared *PbTufM* aRNA with PbWT and PbEV.

The sensitivity of the silenced *PbTufM* aRNA strain to oxidative stress was evaluated by challenged the PbWT, PbEV, and *PbTufM* aRNA cells with hydrogen peroxide (H_2_O_2_). All concentrations of H_2_O_2_ used reduced the viability of *PbTufM* aRNA. When compared to the PbWT, the exposition to 10 mM, 50 mM and 100 mM reduced the viability of *PbTufM* aRNA in 45.6%, 50.5% and 51.7%, respectively; and when compared to PbEV, the viability reduction was 44.4%, 59% and 53.3% respectively (Fig. 7A); indicating that the down expression of PbTUFM resulted in an increase in the sensitive to hydrogen peroxide.

The survival of *PbTufM* aRNA adhered and internalized during the infection of RAW 264.7 macrophages was determined by evaluating the number of colony-forming units (CFUs) 2 h post-infection. PbWT and PbEV showed no significant difference, whereas a reduced number of CFUs were recovered from* PbTufM* aRNA to both conditions, yeasts adhered and internalized (Fig. 7B). This suggests that PbTUFM downexpression resulted in yeasts less able to survive the macrophage environment, maybe due the fact to be more sensitive to oxidative stress, a condition found in the phagocyte environment.

### Knockdown isolate *PbTufM* aRNA has attenuated virulence in *Galleria mellonella* and murine models of paracoccidioidomycosis

#### *G. mellonella* model

**Figure 8:**
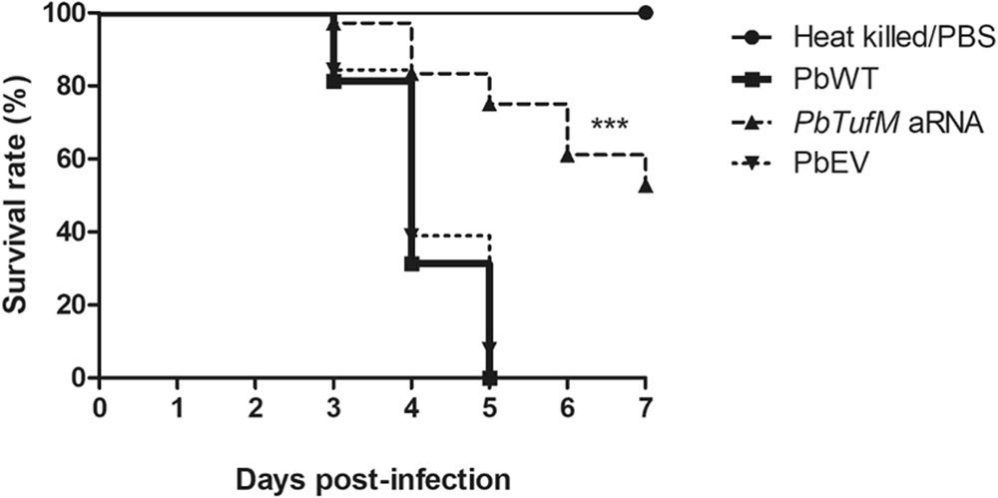
Virulence of *P. brasiliensis* in *Galleria mellonella* infection model decreases in silenced *PbTufM* aRNA isolate. *G. mellonella* survival after infection with 5 × 10^6^ yeast cells from PbWT, PbEV, and *PbTufM* aRNA. Controls groups were injected with PBS and heat-killed yeast cells. *p* < 0.0001 when *PbTufM* aRNA was compared with PbWT and PbEV.

*G. mellonella* larvae were injected with PbWT, *PbTufM* aRNA, or PbEV isolates and incubated at 37 °C for 7 days. As shown in Fig. 8, the *PbTufM* aRNA isolate had a higher rate of larval survival than the PbWT and PbEV isolates. The percentage of surviving larvae 7 days after infection with *PbTufM* aRNA was 52.7%, whilst at the end of the fifth day all larvae infected with PbWT and PbEV were dead. No mortality was observed in PBS-injected or heat-killed yeast cells. These results demonstrate that *PbTufM* aRNA causes significantly less mortality of the larvae, suggesting that the depletion of PbTufM reduces virulence and is important in fungal pathogenesis.

#### Murine model

**Figure 9:**
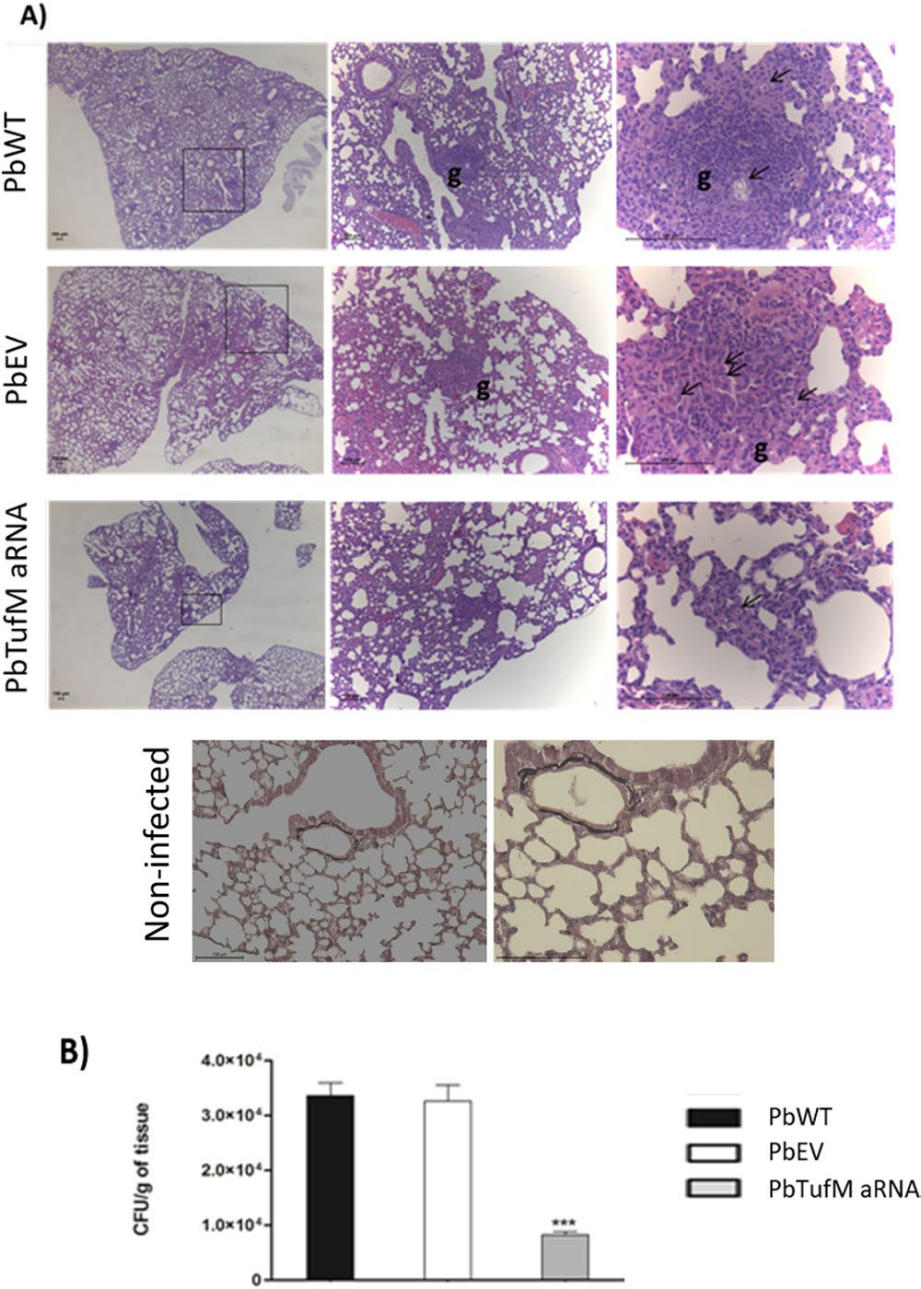
Attenuated virulence of the *PbTufM* aRNA silenced isolate during murine paracoccidioidomycosis. (**A**) Histopathological analysis of mice infected intratrachealy with 3 × 10^5^ yeast cells of PbWT, PbEV, or *PbTufM* aRNA 15 days after infection; g: granulome; the arrows indicate the fungus; and non-infected mice. (**B**) Detection of viable fungal cells of PbWT, PbEV, or *PbTufM* aRNA per gram in lung tissues of mice after 15 days of infection. *p < 0.05, **p < 0.01 and ***p < 0.0001 when comparing *PbTufM* aRNA with PbWT and PbEV. (n = 10 per group).

*PbTufM* aRNA virulence was also evaluated in a murine model of PCM. The level of disease in the lungs of mice infected with *PbTufM* aRNA, PbWT, or PbEV was evaluated by counting the number of CFUs recovered and by histopathological analysis (Fig. 9A). The fungal burden was significantly lower in the lungs of mice infected with *PbTufM* aRNA 15 days post-infection. A 75.6% and 74.5% reduction in fungal burden was observed compared with PbWT and PbEV, respectively (Fig. 9B). The mice inoculated with PbWT, PbEV, or *PbTufM* aRNA cells showed inflammatory responses in the lung tissue that were directly related to fungal spread and proliferation, characterized by the infiltration of bronchovascular bundles by leukocytes and granuloma formation by mononuclear cells surrounded by yeast cells. However, the mice infected with *PbTufM* aRNA had lower numbers of yeast cells and giant cells, and reduced granuloma formation compared to mice infected with PbWT or PbEV, likely due to the reduced number of viable cells (Table 2). The number of granulomas (Table 2) reflected the number of CFUs (Fig. 9B).

**Table 2:** Criteria used in lung histopathological analysis.

Isolates	Mean number of fungi (±SD)	Mean number of granuloma (±SD)	% of lung tissues with giant cells (±SD)
PbWT	1.7 (1.5)	1.7 (0.8)	66% (0.5)
PbEV	2.2 (1.4)	2.2 (0.4)	60% (0.5)
PbTufM aRNA	0.6 (0.8)**	0.9 (0.36)*	40% (0.4)***
PBS	0 (0)	0 (0)	0 (0)

SD: standard deviation. *p < 0.05, **p < 0.01 and ***p < 0.0001 when compared* PbTufM* aRNA with PbWT and PbEV.

## Discussion

Previously, we suggested that the *P. brasiliensis* surface protein TufM is involved in mediating interactions with pneumocytes, host ECM components, and the fibrinolytic system^16^. Here, we constructed a silenced strain using aRNA in order to evaluate the effects of PbTUFM downregulation on *P. brasiliensis* phenotype, fitness, and virulence.

The silenced strain had reduced levels of TufM both on the cell surface and in intracellular compartments. Furthermore, we verified the presence of TufM in the mitochondria of *P. brasiliensis* yeast cells. It has been shown that the EF-Tu protein is localized in the mitochondria of *P. lutzii* yeast cells^27^ by using nanoUPLC-MS^E^ on the mitochondrion-enriched fractions. However, the mechanisms by which this protein is translocated to the cell surface are unclear, especially as the conventional secretion or anchoring signals are unknown. TufM may be translocated non-conventionally by vesicles like other *P. brasiliensis* components^28^.

PbTUFM downregulation did not cause morphological alterations in the yeast cells, which grew normally in a rich fermentative medium *in vitro*, and did not change fungal viability when compared to wild-type and control yeasts. In a chemically defined fermentation medium, *PbTufM* aRNA showed a subtle decrease in growth and viability. A greater difference was observed under respiratory conditions with glycerol as a sole carbon source, suggesting that the silenced strain has impaired respiratory abilities.

Genetic alterations to translation on mito-ribosomes can result in a cell proliferation detriment, probably due to defects associated with aerobic metabolism even though proliferating cells require glycolisys as the main source to their metabolic demands^29^. Mithocondrial EF-Tu null mutants in other species, such as *S. cerevisiae*, are defective for mithochondrial translation, accumulate deleted mtDNA molecules or have total loss of mtDNA^18,30–32^, and have phenotypes with small cell size and slow growth^33,34^. In our study PbTUFM was still expressed, hence the strain construct was not a null mutant, however respiratory-deficient phenotypes similar to those reported previously were observed. The *Cryptococcus neoformans vad1Δ* mutant identified in an insertional mutagenesis screen exhibited decreased expression of TUF1, which encodes an *EF-Tu* homologue. This mutant had a moderate melanization deficiency due to reduced laccase expression, severe virulence attenuation, and fluconazole resistance^23^. In the *vad1Δ* mutant, TUF1 downregulation was associated with impaired growth on a respiratory medium with a glycerol carbon source, suggesting defective mitochondrial function^35^.

The amount and quality of the proteins produced by translation can be affected by the degree of translation elongation. The rate of codon translation is inversely proportional to the occupancy of elongating ribosomes with mRNAs featuring this codon^36^. The silenced strain showed higher puromycin polypeptide incorporation, which may be due to the downregulation of TUFM in *P. brasiliensis* triggering the upregulation of cytosolic proteins. This effect was demonstrated in *Trypanosoma brucei* with mitochondrial EF-TU silencing, where the downregulation of many nuclear-encoded cytochrome oxidase subunits and bc1-complex components resulted in the upregulation of most cytosolic ribosomal proteins^37^. Conversely, the elongation defect in the silenced strain may be due to slower elongation leading to a higher ribosome occupancy which favors the incorporation of puromycin, as there are more nascent polypeptides to incorporate puromycin or more time for this process to occur^38^.

When comparing the interaction (adhesion/invasion) capabilities of the different isolates, TUFM-silenced *P. brasiliensis* had significantly reduced interactions with pneumocytes due to the inhibition of TufM on its surface^16^. This effect was also observed when cell surface TufM was blocked in the silenced strain, with a significant reduction in pneumocyte interactions.

As for the phenotypic differences of *PbTufM* aRNA, when exposed to oxidative stress the silenced strain showed reduced viability, as observed in *S. cerevisiae*^39^. Besides generating energy via oxidative phosphorylation, mitochondria also coordinate several aspects of cellular physiology and functional responses to different stressors^40,41^. Additionally, the capacity of pathogenic fungi to cause disease has been associated with mitochondrial activity, as shown by mitochondrial mutants with attenuated virulence in animal models^42^. The consequences of dysfunctional mitochondria may be related to decreased fitness, metabolic variation, and sensitivity to oxidative stress. Moreover, the environment pressure that operates on respiration-deficient mutants in the host and *in* vitro may be different, can even trigger a compensatory pathways mainly in the host despite the loss of mitochondrial function that contribute to virulence^42^.

The silenced strain also showed reduced survival following interaction with macrophages, as indicated by the lower number of CFUs. During infection, thermally dimorphic fungi such as *Histoplasma capsulatum*, *Blastomyces dermatitidis*, and *P. brasiliensis* are frequently subjected to stresses, including ROS and RNS which are produced by host cells^43–45^. Phagocytosed pathogens find a hostile envinroment in the macrophage, through exposure to ROS and RNS. In order to protect themselves from the harmful effects of ROS, fungal cells have developed strategies to reduce ROS levels^46,47^. Using a proteomics approach, Chaves *et al*.^48^ demonstrated that TufM was upregulated during the infection of alveolar macrophages by *P. brasiliensis*. PbTufM may therefore play a role during the phagocytosis of *P. brasiliensis* by macrophages in response to the stressful of the internal environment of the macrophages.

In the *G. mellonella in vivo* model, the *PbTufM* aRNA strain had significantly reduced virulence compared to the wild-type and empty vector controls. The relationship between greater adhesion to pneumocytes and virulence in *G. mellonella* and murine models of infection, and high levels of adhesin expression in *Paracoccidioides* spp.^49^ have been reported previously.

*PbTufM* aRNA was able to infect the lungs of mice, however PbTUFM downregulation significantly reduced granuloma formation and the presence of fungal cells, whilst giant cells also lowered the fungal burden in the lungs after intratracheal infection.

Mansano *et al*.^50^ showed that the number of *P. brasiliensis* in the lung lesions decreased during the later periods of infection in a manner directly proportional to the lowest number of granulomas. This suggests that granuloma formation at the beginning of the infection could be an initial attempt by the host to prevent or decrease the multiplication and spread of the fungi. Host factors as susceptibility and resistance to infection modulate the granuloma formation in experimental models^51^. However, disease establishment and progression rely on virulence, antigenic profile, environmental conditions, and host immune responses^52^. Macrophages constitute part of the granuloma structure^53^ in the typical inflammatory tissue response to *Paracoccidioides* spp.^54^, which promotes the recognition and processing of specific antigens and plays an essential role in antigen-specific T-cell activation. Isolated macrophages form giant cells with plasmocytes and neutrophils^55,56^. Giant cell formation modulates the secretory and synthetic functions of macrophages, and may occur due to the presence of poorly digestible or indigestible foreign material, or persistent pathogens that are not eliminated for various reasons^57^. We demonstrated that PbTUFM downregulation resulted in more efficient fungal clearance from mouse lungs post-infection, whilst a lower number of granulomas and giant cells indicated reduced inflammation levels, possibly due to the reduced fitness of the strain in the hostile host environment.

Pleiotropy refers to the occurrence of several phenotypes occasioned by a single gene mutation which may occur in yeasts as well as in humans^58,59^. The downexpression of PbTUFM assembling into this strain multiple phenotypes, suggesting that PbTUFM has pleiotropic effects on many biological characteristics of *P. brasiliensis*.

In this study to *PbTufM* aRNA, various phenotypes may be related to the impairment of translation rate, respiratory metabolism and ability to interact with pneumocytes; greater susceptibility to oxidative stress and macrophage killing activity, indicating a link between the PbTUFM expression and virulence with consequences to resistance in the mice. However, we did not know if these multiples observed phenotypes are related specifically to a single or multiple roles of PbTufM, which can also be influenced by the assessed environmental condition. Further studies as transcriptome analysis can help to investigate the pleiotropic effect of PbTufM.

Understanding the virulence factors exploited by human fungal pathogens during infection is of great importance, as their targeted inhibition may in turn reduce fungal pathogenicity. Finally, we suggest that the TufM protein is required for full *P. brasiliensis* virulence, and that future studies should explore the possibility of its use as a target for controlling PCM.

## Methods

### Microorganisms and culture conditions

*P. brasiliensis* strain Pb18 (PbWT, wild type) was cultured in liquid BHI medium with 1% glucose under agitation (150 rpm) at 37 °C, and yeast cells were harvested during the exponential phase (72 h). The Pb18 strain was used for all genetic manipulations^60^. All experiments were performed in the class III biosafety cabinet.

The *Agrobacterium tumefaciens* strain LBA1100^61^, which contains the binary vector pUR5750^62^, was used as a recipient for binary vectors containing cassettes, and was maintained at 28 °C in Luria-Bertani (LB) medium containing kanamycin (100 μg mL^−1^) for selection purposes^63^.

The *E. coli* DH5α strain was used to cloning and propagation of vectors; the bacteria grown at 37 °C on LB medium with kanamycin (50 μg mL^−1^)^64^.

### Constructs for PbTUFM gene silencing

To obtain an isolate with reduced PbTUFM gene expression, we used aRNA and ATMT according to the methods described by Almeida *et al*.^65^. DNA from Pb18 was isolated and the fragment referring to aRNA oligonucleotide targeting the exon coding sequence of PbTUFM (PADG_01949.1; Broad Institute) amplified using a high-fidelity proofreading DNA polymerase^63^. The oligonucleotides used are described in Table 3.

**Table 3:** Primers designed for antisense oligonucleotide and for *hph* PCR amplification, and for RT-qPCR analyses.

Oligonucleotides	forward	reverse
PbTufM	5′ CCGCTCGAGCGGTAAGGCTCCTGAGGAGCGG 3′	5′ GGCGCGCCATCAGCGTGTCCGGGGCAGT 3′
hph	5′ AACTCACCGCGACGTCTGTCGA 3′	5′ CTACACAGCCATCGGTCCAGA 3′
PbTUFM	5′ TATTCGACCGACAAACGACA 3′	5′ TGTTCGCAGCACCAGTAATC 3′
β-tubulin	5′ GTGGACCAGGTGATCGATGT 3′	5′ ACCCTGGAGGCAGTCACA 3′

*Underlined: restriction sites for *Xho*I (forward) and *Asc*I (reverse).

The PbTUFM antisense fragment was inserted into a pCR35 plasmid under the control of the calcium-binding protein gene (*CBP-1*) promoter region from *Histoplasma capsulatum*^66^, and was propagated in *E. coli* DH5α. Each *CBP-*1 promoter-AS cassette was subcloned into a pUR5750 plasmid, the parental binary vector used to harbor the transferred DNA (T-DNA) and an antisense molecule^67^. A recombinant control was generated by transforming Pb18 with an empty vector (PbEV)^65,68^. After transformation, the cells were spread on BHI medium supplemented with hygromycin (100 μg mL^−1^) to select for transformants. Colony formation was monitored at 36 °C for 15–20 days. The phenotypic stability of the transformants was assessed by consecutive (at least three times) subculturing in BHI medium nonselective and then again in medium non-selective and selective medium with hygromycin B (150 μg mL^−1^) for three consecutive times at 36 °C^63,69^.

### Molecular detection of the hygromycin resistance gene (*hph*)

The isolation of genomic DNA from PbWT, PbEV, and *PbTufM* aRNA was performed as described by Van Burik *et al*.^70^. To confirm the transference of the *hph* cassette, PCR amplification was performed to detect the *hph* fragment (~1000 bp) using the primers shown in Table 3. PCR cycling was carried out as described by Marcos *et al*.^71^, and the PCR products analyzed by electrophoresis in 1% agarose gels and visualized using GelRed under UV light.

### Gene expression analysis by real-time PCR

Both, Trizol® Reagent (Invitrogen, Waltham, Massachusetts, USA) and mechanical disruption by maceration with glass beads were employed to extract the total from PbWT, PbEV, and *PbTufM* aRNA cells^60,72^. The first cDNA strand was reverse transcribed using the RevertAid^TM^ H Minus Reverse Transcriptase (Fermentas, Waltham, Massachusetts, USA) with 1 μg of total RNA per reaction. Real-time PCR reactions were carried out in triplicate using specific PbTUFM primers (Table 3) in conjunction with Maxima SYBR Green/ROX qPCR Master Mix 2 × (Fermentas) according to the manufacturer’s instructions on a 7500 Cycler Real-Time PCR System (Applied Biosystems®, Waltham, Massachusetts, USA). The dissociation curves were analyzed for all reactions to verify single peaks/products^73^. Relative quantification values were calculated using the 2^−ΔΔCt^ method with the housekeeping β-tubulin gene (primers in Table 3) as an endogenous control and the target gene expression levels in PbEV as calibrators^74^.

### Microscopic examination of morphology

Yeast cells grown under standard conditions were washed with PBS. A 50 μL suspension of cells in PBS (1 × 10^6^ cells mL^−1^) was stained using 25 mM Calcofluor and observed under a Leica DMLB fluorescence microscope using a 40× objective lens.

### Growth ability using different carbon sources

The growth rates of PbWT, PbEV, and *PbTufM* aRNA cells in liquid BHI (1% glucose, amino acids, and polypeptides as carbon sources), MMcM (1% glucose as a carbon source)^75^, and MMcM (1% glycerol as a carbon source) media (100 mL) were incubated at 37 °C and measured at different times until the stationary phase of growth was reached. Yeast cells were counted every 24 h using a hemocytometer with optical microscopy. All cultures started at an initial concentration of 10^6^ cells mL^−1^, as described by Torres *et al*.^72^.

The length of time required for each strain to double the number of cells (doubling time) during log phase was calculated using the formula of Rieg *et al*.^76^: ln 2 × *t/*(ln *b* − ln *a*) (*t* = time period in hours; *a* = initial cell concentration; *b* = final cell concentration).

Yeast cells were generally diluted to concentrations of 10^5^, 10^6^, and 10^7^ cells in PBS, plated on the culture media described above, and incubated for 7 days at 37 °C.

### Puromycin-labbeling *western blotting*

Firstly, 4-fold serial dilutions of PbWT, PbEV, and *PbTufM* aRNA were prepared, and 5 µL of each dilution (10^5^ to 10^2^) was plated onto BHI plates with different concentrations of puromycin (0, 40, 200, or 1000 µM; Sigma-Aldrich, St. Louis, Missouri, USA). The plates were incubated at 37 °C for 3 days to determine sensitivity to puromycin. Once puromycin sensitivity had been verified, all the strains were grown in a liquid medium until the exponential phase of growth was reached (3 days at 37 °C and 150 rpm). To evaluate the translation rate, the yeast cells were treated with 200 µM of puromycin for 12 h under the conditions described above. Protein extracts were then obtained by alternating freezing in liquid nitrogen and macerating with glass beads according to the methods described by Marcos *et al*.^16^. Protein concentrations were estimated using the Bradford method. Equivalent amounts of protein (50 µg) isolated from all strains after the puromycin treatment were used for western blotting with the anti-puromycin antibody, and Coomassie staining and Ponceau red loading dye as controls. Nascent polypeptides containing puromycin were detected using a secondary antibody conjugated to alkaline phosphatase according to the manufacturer’s instructions. Translation rate was estimated based on the intensity of immunoreactive bands using Image-J (Bethesda, Maryland, USA).

### Mitochondrial location of TufM in *P. brasiliensis*

To determine whether TufM was also localized in the mitochondria of yeast cells grown in Fava-Netto medium^77^, immunochemistry was carried out at the ultra-structural level by immunogold labeling, as described by Marcos *et al*.^16^, using a polyclonal anti-PbTufM antibody previously obtained by our group.

### TufM quantification by flow-cytometry and western blot analysis of *P. brasiliensis* cell-wall associated proteins

To determine whether silencing affected the cytosolic and cell surface levels ofTufM, we performed the assay described by Silva *et al*.^78^ with modifications. PbWT, PbEV, and *PbTufM* aRNA cells were grown in BHI medium as already described. Two aliquots of each strain (10^7^ cells) were collected. The first aliquot (non-permeabilized cells) was fixed in 3.5% paraformaldehyde for 20 min at room temperature (25 °C), washed twice with PBS, and incubated with blocking solution (BSA-bovine serum albumin 2%) for 1 h. After washing, the aliquot was incubated with polyclonal anti-PbTufM (diluted 1:10 in blocking solution) at 4 °C overnight. The second aliquot (permeabilized cells) was fixed as described above, washed, and permeabilized with Triton-X 100 (diluted 1:100 in PBS) for 45 min at room temperature. After washing, the aliquot was incubated with polyclonal anti-PbTufM as described above. After multiple washes in PBS, the cells of both aliquots were incubated with anti-rabbit IgG labeled with phycoerythrin (PE) (diluted 1:10 in PBS) (Sigma-Aldrich) for 2 h at room temperature. After two washes, the fluorescence intensity was estimated by cytometry (FACSCanto^TM^, Becton Dickinson, East Rutherford, New Jersey, USA), counting 10^4^ cells. Both aliquots were also incubated with pre-immune rabbit serum and with the secondary antibody only as controls and were observed using an IN Cell Analyzer 2000 (GE Healthcare, Chicago, Illinois, USA).

The cell surface associated proteins were extracted using dithiothreitol (DTT), a reducing agent^79^. According to Longo *et al*.^80^, the yeast cells were harvested by centrifugation (6,000 rpm, 15 min), washed with ice-cold buffer containing 25 mM Trish-HCl, pH 8.5 (the same buffer used in the subsequent steps), vigorously vortexing for 1 min between each wash, and then treated with 2 mM DTT-buffer solution containing 5 mM ethylenediamine tetraacetic acid (EDTA) and 1 mM phenyl methyl sulphonyl (PMSF). The samples were incubated at 4 °C with gentle agitation. By centrifugation the cells were pelleted and the supernatant containing the DTT-extractable proteins were filtered using a 0.22-µ filters followed by dialysis in Amicon (Millipore) with 0.1 M ammonium acetate. After the sample lyophilization, the surface proteins extracts were suspended in deionized water and precipitated with acetone at −70 °C; the precipitated proteins separated by centrifugation (10,000 rpm a 4 °C) were washed with acetone and dried in the air. Protein concentration was estimated using the Bradford method. Equivalent amounts of PbEV and *PbTufM* aRNA proteins were separated by gel electrophoresis using SDS-PAGE^81^ and were transferred onto nitrocellulose membranes (Hybond-C extra)^64^. The membranes were blocked with 5% skim milk and 1% bovine serum (BSA) for 4 h at room temperature and washed three times with PBST (PBS with 0.05% Tween-20). The membranes were then incubated with primary polyclonal antibodies, anti-GAPDH (diluted 1:1000; Invitrogen) and anti-PbTufM^16^ (diluted 1:250; in blocking buffer overnight at room temperature and were washed as described above. Blots were developed using a horseradish peroxidase-labelled second antibody against rabbit IgG (diluted 1:200 in blocking buffer; Sigma®) with 3,3′ diaminobenzidine as the substrate. Relative levels of PbTufM were determined by band quantification using ImageJ software (NIH), and were normalized to GAPDH, a protein abundantly founded in the *P. brasiliensis* cell-wall^82^, which was used as an internal control.

### Interaction between *P. brasiliensis* cells and host cells

A549 cells (10^5^ cells per well) were added to HAM-F12 medium (Cultilab, Campinas, São Paulo, Brazil) supplemented with 10% fetal bovine serum in 96-well culture plates and incubated at 37 °C in 5% CO_2_. Monolayer formation was confirmed by microscopy. Cells were washed and infected with PbWT, PbEV, or *PbTufM* aRNA (10^5^ cells per well, multiplicity of infection (MOI): 1:2 yeast:pneumocytes) previously stained for 30 min at 37 °C with 10 μM of CFSE (5,6-carboxyfluorescein diacetate N-succinimidyl ester) which stains the fungal cell wall, and were then washed and resuspended in HAM-F12 medium. Triplicate wells were arranged on each plate and four plates were set up to perform a time course analysis of infection (at 2, 5 and 8 h) under the same conditions as described above. After infection, unbound yeast cells were removed by washing three times with PBS. The bound cells were detached from the plastic with cold PBS and a rubber cell scraper, put on ice, fixed with 4% paraformaldehyde, and analyzed by flow-cytometry using a FACSCantoTM (Becton Dickinson). Uninfected host cells and unlabeled yeast cells were used as internal controls to define the gates and to evaluate autofluorescence. The excitation wavelength was 488 nm, and emitted light was collected using the 530/30 nm band-pass filter. Data were processed and analyzed using FACs-Diva software. A549 cells were gated and the mean CFSE fluorescence (corresponding to labeled yeast cells) of this gate was used to quantify the association between *P. brasiliensis* and host cells^60^.

The strains were also treated with anti-PbTufM as described previousl^16^ and labelled with CFSE before A549 infection to verify whether blocking cell surface PbTufM altered the association between A549 and *PbTufM* aRNA cells. Analysis was performed as above.

### Viability assay after oxidative stress and *P. brasiliensis* macrophage infection

Individual inoculums of PbWT, PbEV, and *PbTufM* aRNA cells (100 μL, 10^6^ cells mL^−1^) were treated with different H_2_O_2_ concentrations (10, 50, and 100 mM) for 1 h. The cells were then pelleted by centrifugation, washed twice, resuspended with PBS and plated on solid BHI to evaluate the viability counting the colony forming units (CFU) after 7 days of incubation at 37 °C.

RAW 264.7 macrophages were used in the *P. brasiliensis* survival assay, cultured in Dulbecco´s Modified Eagle Medium (DMEM) containing 10% bovine fetal serum at 37 °C and 5% CO_2_ until completely confluent. A total of 2.5 × 10^4^ RAW macrophages was added to each well of 96-well polypropylene plates with DMEM medium and incubated for 2 h at 37 °C and 5% CO_2_. Then 2.5 × 10^4^ PbWT, PbEV, and* PbTufM* aRNA cells were added to each well with the macrophages, resulting in a yeast:macrophage cell ratio of 1:2, since the doubling time for RAW cells is around 15 h. The cells were incubated for 2 h at 36 °C and 5% CO_2_, then washed three times with PBS to remove non-adherent yeast cells. To kill only extracellular fungi, the cells were treated with 15 µg mL^−1^ of ketoconazole solution in DMEM medium for 1 h at 36 °C^10,83^. The macrophages were lysed with 100 µL of MilliQ-water, the fungal cells were recovered and plated onto BHI with 1.5% glucose, 5% *P. brasiliensis* (strain 192)-spent culture medium, 4% bovine fetal serum and gentamycin (40 mg L^−1^)^84^; the same was done without ketoconazole treatment to determine the total rate of infection (adhered/internalized yeasts); the CFUs were counted after 14 days of incubation at 37 °C.

### *Galleria mellonella* infection model

Sixteen *G. mellonella* larvae (0.1–0.2 g) were selected for inoculation. Each larva was cleaned with 70% ethanol and, using a Hamilton syringe (Hamilton, Nevada, USA), was injected through the last left proleg with 10 μL (5 × 10^6^ cells) of PbWT, PbEV, or *PbTufM* aRNA cell suspensions prepared in PBS without cell clumps. Each experiment was repeated independently three times. Control groups were inoculated with a) PBS, to monitor the effects of physical injury on survival; b) heat-killed *P. brasiliensis* yeast cells, to evaluate the effects of osmotic stress due to the inoculum size on survival, and c) non-infected larvae. The larvae were then incubated in Petri dishes at 37 °C for 7 days and checked daily for mortality^85^.

### Animal use and ethics statement

Pathogen-free mice were obtained from the animal facilities of the University of São Paulo, Brazil. The experiments were approved by the Ethics Committee on Animal Experimentation of the Faculty of Pharmaceutical Sciences of Araraquara – UNESP (Proc. 47/2016/CEUA/FCF). Animal experiments was conducted in strict accordance with Brazilian Federal Law 11,794 establishing procedures and the state law establishing the Animal Protection Code of the State of São Paulo.

### Intratracheal infection of BALB/c mice

We used male BALB/c mice (6–8 weeks-old), which were anesthetized intraperitoneally with 200 μL of a xylazine (10 mg kg^−1^) and ketamine (80 mg kg^−1^) solution. Viable PbWT, PbEV, or *PbTufM* aRNA cells (3 × 10^5^ cells) were administered to each mouse by intratracheal inoculation with a 26-gauge needle after hyperextension of the neck and exposition of the trachea at the level of the thyroid. Incisions were sutured with 5-0 silk^86^.

### Fungal burden in lungs of infected mice

The lungs were removed, weighed, and homogenized in 1 mL of PBS at 15 days post-infection. Samples (100 μL) were then plated on solid BHI medium supplemented as described above. Petri dishes were incubated at 37 °C and colonies counted after 20 days.

### Histopathology

Formalin (10%)-fixed murine lungs were embedded in paraffin, sectioned, and stained with hematoxylin and eosin (H&E). Microscopic analysis was performed and evaluated blind by a pathologist using a ZEISS Primo Star (Zeiss, Oberkochen, Germany) microscope with 4 and 40× objectives. The number of fungi and granulomas, and the percentage of lung tissues with giant cells were recorded

### Statistical analysis

Statistical comparisons were made using analysis of variance (one-way ANOVA) followed by Tukey’s test, and Student’s *t-*tests. For the survival curve, statistical analyses of the results of independent experiments were performed using the Long-rank test. *p-*values of <0.05 were considered statistically significant.

## References

[1] Brummer E, Castaneda E, Restrepo A (1993). Paracoccidioidomycosis: an update. Clin Microbiol Rev.

[2] Colombo AL, Tobón A, Restrepo A, Queiroz-Telles F, Nucci M (2011). Epidemiology of endemic systemic fungal infections in Latin America. Med Mycol.

[3] Martinez R (2015). Epidemiology of Paracoccidioidomycosis. Rev Inst Med Trop Sao Paulo.

[4] Turissini DA, Gomez OM, Teixeira MM, McEwen JG, Matute DR (2017). Species boundaries in the human pathogen Paracoccidioides. Fungal Genet Biol.

[5] Loth, E. A. *et al*. Infection caused by the yeast form of Paracoccidioides brasiliensis. *In JMM Case Reports* **1** (2015).

[6] Tatsumi Y (2013). Mechanism of action of efinaconazole, a novel triazole antifungal agent. Antimicrob Agents Chemother.

[7] de Groot PW, Bader O, de Boer AD, Weig M, Chauhan N (2013). Adhesins in human fungal pathogens: glue with plenty of stick. Eukaryot Cell.

[8] Croft CA, Culibrk L, Moore MM, Tebbutt SJ (2016). Interactions of Aspergillus fumigatus Conidia with Airway Epithelial Cells: A Critical Review. Front Microbiol.

[9] Mendes-Giannini MJ (2006). Binding of extracellular matrix proteins to Paracoccidioides brasiliensis. Microbes Infect.

[10] Mendes-Giannini MJ (2004). Invasion of epithelial mammalian cells by Paracoccidioides brasiliensis leads to cytoskeletal rearrangement and apoptosis of the host cell. Microbes Infect.

[11] Camacho E, Niño-Vega GA (2017). Paracoccidioides Spp.: Virulence Factors and Immune-Evasion Strategies. Mediators Inflamm.

[12] Marcos CM (2016). Anti-Immune Strategies of Pathogenic Fungi. Front Cell Infect Microbiol.

[13] da Silva, J. F. *et al*. Advances and challenges in paracoccidioidomycosis serology caused by Paracoccidioides species complex: an update. *Diagn Microbiol Infect Dis* (2015). 10.1016/j.diagmicrobio.2015.06.004 26494541

[14] Ruiz OH (2011). Alternative oxidase mediates pathogen resistance in Paracoccidioides brasiliensis infection. PLoS Negl Trop Dis.

[15] Vale PF (2016). Beyond killing: Can we find new ways to manage infection?. Evol Med Public Health.

[16] Marcos, C. M. *et al*. Identification and characterization of elongation factor Tu (EF-Tu), a novel protein involved in Paracoccidioides brasiliensis-host interaction. *FEMS Yeast Res* (2016). 10.1093/femsyr/fow079 27634774

[17] Christian BE, Spremulli LL (2012). Mechanism of protein biosynthesis in mammalian mitochondria. Biochim Biophys Acta.

[18] Chiron S, Suleau A, Bonnefoy N (2005). Mitochondrial translation: elongation factor tu is essential in fission yeast and depends on an exchange factor conserved in humans but not in budding yeast. Genetics.

[19] Clark BF, Nyborg J (1997). The ternary complex of EF-Tu and its role in protein biosynthesis. Curr Opin Struct Biol.

[20] Souza DP (2005). Paracoccidioides brasiliensis translation and protein fate machineries revealed by functional genome analysis. Genet Mol Res.

[21] Rosenthal LP, Bodley JW (1987). Purification and characterization of Saccharomyces cerevisiae mitochondrial elongation factor Tu. J Biol Chem.

[22] Francisci S, Montanari A (2017). Mitochondrial diseases: Yeast as a model for the study of suppressors. Biochim Biophys Acta Mol Cell Res.

[23] Panepinto JC (2010). Overexpression of TUF1 restores respiratory growth and fluconazole sensitivity to a Cryptococcus neoformans vad1Delta mutant. Microbiology.

[24] Ellis D (2002). Amphotericin B: spectrum and resistance. J Antimicrob Chemother.

[25] Hernández, O. *et al*. The hydrolase PbHAD32 participates in the adherence of Paracoccidioides brasiliensis conidia to epithelial lung cells. *Med Mycol* (2011). 10.3109/13693786.2011.619583 21988701

[26] Mendes-Giannini MJ (2004). Invasion of epithelial mammalian cells by Paracoccidioides brasiliensis leads to cytoskeletal rearrangement and apoptosis of the host cell. Microbes Infect.

[27] Casaletti L (2017). Analysis of Paracoccidioides lutzii mitochondria: a proteomic approach. Yeast.

[28] Peres da Silva R (2015). Extracellular vesicles from Paracoccidioides pathogenic species transport polysaccharide and expose ligands for DC-SIGN receptors. Sci Rep.

[29] Battersby BJ, Richter U (2013). Why translation counts for mitochondria - retrograde signalling links mitochondrial protein synthesis to mitochondrial biogenesis and cell proliferation. J Cell Sci.

[30] Myers AM, Pape LK, Tzagoloff A (1985). Mitochondrial protein synthesis is required for maintenance of intact mitochondrial genomes in Saccharomyces cerevisiae. The EMBO journal.

[31] Chen XJ, Clark-Walker GD (2000). The petite mutation in yeasts: 50 years on. Int Rev Cytol.

[32] Contamine V, Picard M (2000). Maintenance and integrity of the mitochondrial genome: a plethora of nuclear genes in the budding yeast. Microbiol Mol Biol Rev.

[33] Jorgensen P, Nishikawa JL, Breitkreutz BJ, Tyers M (2002). Systematic identification of pathways that couple cell growth and division in yeast. Science.

[34] Yoshikawa K (2011). Comprehensive phenotypic analysis of single-gene deletion and overexpression strains of Saccharomyces cerevisiae. Yeast.

[35] Panepinto J (2005). The DEAD-box RNA helicase Vad1 regulates multiple virulence-associated genes in Cryptococcus neoformans. J Clin Invest.

[36] Stadler M, Fire A (2011). Wobble base-pairing slows *in vivo* translation elongation in metazoans. RNA.

[37] Cristodero M (2013). Mitochondrial translation factors of Trypanosoma brucei: elongation factor-Tu has a unique subdomain that is essential for its function. Mol Microbiol.

[38] Schuller AP, Wu CC, Dever TE, Buskirk AR, Green R (2017). eIF5A Functions Globally in Translation Elongation and Termination. Mol Cell.

[39] Valente L (2007). Infantile encephalopathy and defective mitochondrial DNA translation in patients with mutations of mitochondrial elongation factors EFG1 and EFTu. Am J Hum Genet.

[40] Calderone R, Li D, Traven A (2015). System-level impact of mitochondria on fungal virulence: to metabolism and beyond. FEMS Yeast Res.

[41] Green DR, Galluzzi L, Kroemer G (2014). Cell biology. Metabolic control of cell death. Science.

[42] Shingu-Vazquez M, Traven A (2011). Mitochondria and fungal pathogenesis: drug tolerance, virulence, and potential for antifungal therapy. Eukaryot Cell.

[43] Medoff G, Painter A, Kobayashi GS (1987). Mycelial- to yeast-phase transitions of the dimorphic fungi Blastomyces dermatitidis and Paracoccidioides brasiliensis. J Bacteriol.

[44] Johnson CH, Prigge JT, Warren AD, McEwen JE (2003). Characterization of an alternative oxidase activity of Histoplasma capsulatum. Yeast.

[45] Brummer E, Hanson LH, Restrepo A, Stevens DA (1989). Intracellular multiplication of Paracoccidioides brasiliensis in macrophages: killing and restriction of multiplication by activated macrophages. Infect Immun.

[46] Martins VP (2011). Involvement of an alternative oxidase in oxidative stress and mycelium-to-yeast differentiation in Paracoccidioides brasiliensis. Eukaryot Cell.

[47] Gessler NN, Aver’yanov AA, Belozerskaya TA (2007). Reactive oxygen species in regulation of fungal development. Biochemistry (Mosc).

[48] Chaves, E. G. A. *et al*. Proteomic analysis of Paracoccidioides brasiliensis during infection of alveolar macrophage primed or not by interferon gamma. *Frontiers in Microbiology* (2019). 10.3389/fmicb.2019.00096 PMC6371752 30804901

[49] de Oliveira, H. C. *et al*. Importance of adhesins in virulence of Paracoccidioides spp. *Frontiers in Microbiology* **6** (2015). 10.3389/fmicb.2015.00303 PMC4392702 25914695

[50] Mansano ES (2014). Correlation between histopathological and FT-Raman spectroscopy analysis of the liver of Swiss mice infected with Paracoccidioides brasiliensis. PLoS One.

[51] Durbeej M (2010). Laminins. Cell Tissue Res.

[52] Souza PV, Pinto WB, Matas SL (2014). Paracoccidioidomycosis: a rare cause of infectious encephalomyelopathy. Arq Neuropsiquiatr.

[53] Roitt, I., Brostoff, J. & Male, D. Hypersensitivity– Type IV. In *Immunology* 341–348 (MosbyInternational Ltd., London, 1998).

[54] Datta K, Hamad M (2015). Immunotherapy of Fungal Infections. Immunol Invest.

[55] Da Silva FC (2009). Morphologic organization of pulmonary granulomas in mice infected with Paracoccidioides brasiliensis. Am J Trop Med Hyg.

[56] Kaminagakura E, Bonan PR, Jorge J, Almeida OP, Scully C (2007). Characterization of inflammatory cells in oral paracoccidioidomycosis. Oral Dis.

[57] Quinn MT, Schepetkin IA (2009). Role of NADPH oxidase in formation and function of multinucleated giant cells. J Innate Immun.

[58] Dudley, A. M., Janse, D. M., Tanay, A., Shamir, R. & Church, G. M. A global view of pleiotropy and phenotypically derived gene function in yeast. *Molecular systems biology* **1**, 2005.0001-2005.0001 (2005). 10.1038/msb4100004 PMC1681449 16729036

[59] Brunner HG, van Driel MA (2004). From syndrome families to functional genomics. Nat Rev Genet.

[60] Maria Marcos C (2016). Decreased expression of 14-3-3 in Paracoccidioides brasiliensis confirms its involvement in fungal pathogenesis. Virulence.

[61] Beijersbergen A, Dulk-Ras AD, Schilperoort RA, Hooykaas PJ (1992). Conjugative Transfer by the Virulence System of Agrobacterium tumefaciens. Science.

[62] de Groot MJ, Bundock P, Hooykaas PJ, Beijersbergen AG (1998). Agrobacterium tumefaciens-mediated transformation of filamentous fungi. Nat Biotechnol.

[63] Almeida AJ (2007). Towards a molecular genetic system for the pathogenic fungus Paracoccidioides brasiliensis. Fungal Genetics and Biology.

[64] Sambrook, J., Fritsch, E. F. & Maniatis, T. *Molecular Cloning: A Laboratory Manual*, (New York, 1998).

[65] Green, D. R. & Llambi, F. Cell Death Signaling. *Cold Spring Harb Perspect Biol* **7** (2015). 10.1101/cshperspect.a006080 PMC4665079 26626938

[66] Rappleye CA, Engle JT, Goldman WE (2004). RNA interference in Histoplasma capsulatum demonstrates a role for alpha-(1,3)-glucan in virulence. Mol Microbiol.

[67] Torres I (2014). Paracoccidioides brasiliensis PbP27 gene: knockdown procedures and functional characterization. FEMS Yeast Research.

[68] El Ridi R, Tallima H (2009). Schistosoma mansoni *ex vivo* lung-stage larvae excretory-secretory antigens as vaccine candidates against schistosomiasis. Vaccine.

[69] Tamayo D (2017). Paracoccidioides spp. catalases and their role in antioxidant defense against host defense responses. Fungal Genetics and Biology.

[70] van Burik JA, Schreckhise RW, White TC, Bowden RA, Myerson D (1998). Comparison of six extraction techniques for isolation of DNA from filamentous fungi. Med Mycol.

[71] Marcos, C. M. *et al*. Decreased expression of 14-3-3 in *Paracoccidioides brasiliensis* confirms its involvement in fungal pathogenesis. *Virulence* (2015). 10.1080/21505594.2015.1122166 PMC4994830 26646480

[72] Torres I (2013). Inhibition of PbGP43 expression may suggest that gp43 is a virulence factor in Paracoccidioides brasiliensis. PloS one.

[73] Cleary IA, MacGregor NB, Saville SP, Thomas DP (2012). Investigating the Function of Ddr48p in Candida albicans. Eukaryotic Cell.

[74] Livak KJ, Schmittgen TD (2001). Analysis of relative gene expression data using real-time quantitative PCR and the 2(−Delta Delta C(T)) Method. Methods.

[75] Restrepo A, Jiménez BE (1980). Growth of Paracoccidioides brasiliensis yeast phase in a chemically defined culture medium. J Clin Microbiol.

[76] Rieg G (1999). Unanticipated heterogeneity in growth rate and virulence among Candida albicans AAF1 null mutants. Infect Immun.

[77] de B, Netto CF (1963). Disseminated experimental South American blastomycosis of the guinea pig; a pathologic and immunologic study. Pathol Microbiol (Basel).

[78] Silva RC (2014). Extracellular enolase of Candida albicans is involved in colonization of mammalian intestinal epithelium. Front Cell Infect Microbiol.

[79] Klis FM, de Jong M, Brul S, de Groot PWJ (2007). Extraction of cell surface-associated proteins from living yeast cells. Yeast.

[80] Longo LVG, da Cunha JPC, Sobreira TJP, Puccia R (2014). Proteome of cell wall-extracts from pathogenic Paracoccidioides brasiliensis: Comparison among morphological phases, isolates, and reported fungal extracellular vesicle proteins. EuPA Open Proteomics.

[81] Laemmli UK (1970). Cleavage of Structural Proteins during the Assembly of the Head of Bacteriophage T4. Nature.

[82] Barbosa MS (2006). Glyceraldehyde-3-phosphate dehydrogenase of Paracoccidioides brasiliensis is a cell surface protein involved in fungal adhesion to extracellular matrix proteins and interaction with cells. Infection and immunity.

[83] Hanna SA, Monteiro da Silva JL, Giannini MJSM (2000). Adherence and intracellular parasitism of Paracoccidioides brasiliensis in Vero cells. Microbes and Infection.

[84] Singer-Vermes LM, Ciavaglia MC, Kashino SS, Burger E, Calich VL (1992). The source of the growth-promoting factor(s) affects the plating efficiency of Paracoccidioides brasiliensis. J Med Vet Mycol.

[85] Scorzoni L (2015). Comparison of virulence between Paracoccidioides brasiliensis and Paracoccidioides lutzii using Galleria mellonella as a host model. Virulence.

[86] Bueno RA (2016). Antibodies Against Glycolipids Enhance Antifungal Activity of Macrophages and Reduce Fungal Burden After Infection with Paracoccidioides brasiliensis. Front Microbiol.

